# Comprehensive immune cell analysis of human menstrual-blood-derived stem cells therapy to concanavalin A hepatitis

**DOI:** 10.3389/fimmu.2022.974387

**Published:** 2022-09-29

**Authors:** Fen Zhang, Linxiao Fan, Qiuhong Liu, Shima Tang, Sainan Zhang, Lanlan Xiao, Lingjian Zhang, Qian Li, Nueraili Maihemuti, Lanjuan Li

**Affiliations:** ^1^ State Key Laboratory for Diagnosis and Treatment of Infectious Diseases, National Clinical Research Center for Infectious Diseases, Collaborative Innovation Center for Diagnosis and Treatment of Infectious Diseases, The First Affiliated Hospital, Zhejiang University School of Medicine, Hangzhou, China; ^2^ Department of Infectious Disease, Shulan (Hangzhou) Hospital Affiliated to Zhejiang Shuren University, Shulan International Medical College, Hangzhou, China

**Keywords:** autoimmune hepatitis, concanavalin A, menstrual blood-derived stem cells, CyTOF, mesenchymal stem cell therapy

## Abstract

Autoimmune hepatitis is an autoimmune disease with increasing occurrence worldwide. The most common and convenient mouse model is the concanavalin A (ConA) mouse model. Human menstrual-blood-derived stem cells (MenSCs) have shown great potential as a type of mesenchymal stem cell for treating various diseases. Time-of-flight mass cytometry was performed in phosphate-buffered saline control (NC) group and ConA injection with or without MenSCs treatment groups, and conventional flow cytometry was used for further validation. The serum alanine aminotransferase (ALT) and aspartate aminotransferase (AST) levels and H&E staining depicted that MenSCs treatment could significantly alleviate ConA-induced hepatitis. The t-distributed stochastic neighbor embedding (t-SNE) analysis of nine liver samples displayed favorable cell clustering, and the NC group was significantly different from the other two groups. The proportions of CD69^+^ T cells, NKT cells, and PD-L1^+^ macrophages were notably increased by ConA injection, while MenSCs could decrease ConA-induced macrophage percentage and M1 polarization in the liver tissue. The analysis of proinflammatory factors carried out by cytometric bead array demonstrated that tumor necrosis factor alpha (TNF-α), interleukin (IL)-17A, IL-12p70, IL-6, IL-2, IL-1b, and interferon gamma (IFN-γ) were upregulated after ConA injection and then rapidly decreased at 12 h. MenSCs also played an important role in downregulating these cytokines. Here, we described the comprehensive changes in leukocytes in the liver tissue of ConA-induced hepatitis at 12 h after ConA injection and found that MenSCs rescued ConA-induced hepatitis mostly by inhibiting macrophages and M1 polarization in mouse liver.

## Introduction

Autoimmune hepatitis (AIH) is a hepatocytes-specific autoimmune disease that appears among all age groups with an increasing trend (1–3). Histological analysis showed that inflammatory cells infiltrated and surrounded the portal area (4). The infiltrated inflammatory cells mainly included T and B lymphocytes and mature plasma cells. The disease can gradually appear as progressive hepatitis and fibrosis and can even progress to liver cirrhosis and liver cancer (5–7). Although autoimmune liver disease is a chronic liver disease, it can usually develop from acute hepatitis or even fulminant liver failure, manifesting as jaundice and elevated transaminases (5). Clinically, patients with AIH usually receive immunosuppressive treatment using corticosteroids and azathioprine. However, not all patients respond well to immunosuppressive treatment, and this can lead to the development of drug resistance (8).

Mesenchymal stem cells (MSCs), as the most common cell source for stem cell therapy, play an important role in modulating immune responses and have been thoroughly studied for the treatment of autoimmune and inflammatory diseases (9, 10). MSCs are a heterogeneous subset of stromal stem cells first isolated from the bone marrow by Friedenstein *et al.* (11). MSCs can also be obtained from other adult tissues, such as adipose and umbilical cord blood (12–14). Human menstrual-blood-derived stem cells (MenSCs) are a novel MSC type derived from the endometrium, which makes them a source of MenSCs with practical clinical applications (15). MenSCs have been studied for many years for their anti-inflammatory effects by Xiang *et al.* and our research group (16–19). Previous articles reported that key paracrine factors such as stem cell factor (SCF) (20), fibroblast growth factor 21 (FGF21) (21), tumor growth factor (TGF)-β1/2/3, interleukin (IL)-10, and monocyte chemoattractant protein-1 (MCP-1) (22) could be important candidates for MSCs against cell injury and suppressing immune reactions.

AIH mouse models can be clustered into two categories: T-cell tolerance-related models and antigen-related models (23). Among all AIH mouse models, the concanavalin A (ConA)-induced AIH mouse model is still the most widely used. Here, we used the ConA model to imitate an acute state of AIH in our study. To better understand the role of MenSC therapy in AIH immune regulation, single-cell time-of-flight mass spectrometry (CyTOF), conventional flow cytometry, and cytometric bead array were applied.

## Materials and methods

### MenSCs culture and identification

The MenSCs were kindly provided by Charlie Xiang’s lab. Menstrual blood was obtained from three young healthy female volunteers (age under 30 years old) after giving informed consent. Menstrual blood was collected and separated by using Ficoll–Paque to get the interlayer cells. The cells from the third to the eighth passage were used and cultured in Dulbecco’s modified Eagle’s medium (DMEM)/F12 medium with 10% fetal bovine serum (FBS) (Gibco, USA). In addition, the MenSCs had been identified by flow cytometry and can be induced to differentiate to adipocytes, chondroblasts, and osteoblasts (16, 24).

### ConA-induced hepatitis and MenSCs transplantation

C57BL/6 mice (4–6 weeks old, male) were obtained from the Experimental Animal Center of Zhejiang Academy of Medical Sciences. All the mice were acclimatized at the Laboratory Animal Center for 1 week. Mice (20–25 g) were intravenously injected with ConA (15 mg/kg) to establish the AIH model (Sigma-Aldrich, USA). A total of 5 × 10^5^ MenSCs in 0.2 ml phosphate-buffered saline (PBS) (CM group) or an equal volume of PBS (ConA group) was injected through tail veins at the same time point of ConA injection to evaluate the therapeutic effect of MenSCs in AIH. The control group was injected with only PBS (NC group) or MenSCs (NC+MenSCs group). Mice were anesthetized using isoflurane and sacrificed. The serum and liver tissues were harvested at 6, 12, and 24 h in ConA and CM groups. NC and NC+MenSCs groups were harvested at 24 h.

### Evaluation of liver injury

Alanine aminotransferase (ALT) and aspartate aminotransferase (AST) were analyzed following the manual instructions (Nanjing Jiancheng Bioengineering Institute, China). Fixed liver tissues were processed and embedded in tissue embedding cassettes. Three- to five-micrometer thick slices were stained with hematoxylin and eosin (H&E).

### ELISA analysis

The serum mouse IL-10 and human MCP-1 (Beijing Dakwei Biotechnology, China) concentrations were processed with commercial ELISA kits, and OD450 values were measured with a microplate reader (Bio-Rad, USA).

### CyTOF

#### Single-cell suspension preparation

Liver samples were collected from the NC, ConA, and CM groups at 12 h after injection (n = 3 per group). Mouse liver tissues were cut into small pieces and digested with a Mouse Liver Dissociation Kit (Miltenyi Biotec). The cells were filtered through a 70-μm cell strainer, and red blood cells were lysed with ACK lysis buffer. The cell suspension was counted with 0.4% trypan blue solution.

#### Mass cytometry, staining, and data acquisition

The purified antibodies used for mass cytometric analysis are summarized in **Supplementary Table S1**. The cells were washed once with 1× PBS and stained with 250 nM cisplatin (Fluidigm) for 5 min on ice to exclude dead cells. Then, the cells were incubated in Fc receptor blocking solution before staining with a surface antibody cocktail for 30 min on ice. Cells were washed twice with FACS buffer [0.5% bovine serum albumin (BSA)] and fixed in 200 μl intercalation solution (Maxpar Fix and Perm Buffer containing 250 nM 191/193Ir; Fluidigm) overnight. After fixation, the cells were washed once with FACS buffer and then with permeabilization buffer (eBioscience) and stained with intracellular antibody cocktail for 30 min on ice. Cells were washed and resuspended in deionized water with an additional 20% EQ beads (Fluidigm) and then detected by mass cytometry (Helios; Fluidigm).

### CyTOF data analysis

Raw data from each sample were de-barcoded using a doublet-filtering scheme with unique mass-tagged barcodes. The metal ion signal of each channel is similar to each fluorescence intensity of conventional flow cytometry. The original signal obtained on machine needs to be converted by ArcsinH (X/5) before analysis. Each fcs file generated from different batches was normalized using a bead normalization method. Data were manually gated using FlowJo software to exclude debris, dead cells, and doublets, leaving single live immune cells, and CD45^+^ cells were gated for further analysis. The X-shift clustering algorithm was applied to partition the cells into distinct phenotypes based on marker expression levels. The cell type of each cluster was annotated according to the marker expression pattern on a cluster versus marker heatmap. The t-distributed stochastic neighbor embedding (t-SNE) dimensionality reduction algorithm was used to visualize the high-dimensional data in two dimensions and to show the distribution of each cluster and marker expression and differences among the groups or different sample types. The frequencies of the annotated cell populations were analyzed using the t-test.

### Cytometric bead array kit and conventional flow cytometry analysis

CBA-enhanced sensitivity flex sets were used to determine the serum concentrations of TNF-α, IL-17A, IL-12p70, IL-6, IL-2, IL-1b, and IFN-γ (BD Biosciences, USA) following the manufacturer’s instructions.

Spleen tissues were collected and washed with cold PBS. A 70-µm filter was placed on a 50-ml centrifuge tube, cold PBS was added while grinding, the grinding solution was centrifuged at 1,000 rpm for 5 min, and the supernatant was discarded. Cells were washed twice with cold PBS, 2 ml of erythrocyte lysate (Solarbio, China) was added, and the liquid was allowed clear. The reaction was stopped by adding approximately 10 ml of PBS, and the cells were washed with cold PBS and filtered with a 40-µm cell strainer for staining. After the liver tissue was minced, it was put into a 50-ml centrifuge tube, and 5 ml PBS was added with collagenase IV, DNase I, and dispase II and shook with 150 rpm at 37°C for 1 h. The cells were filtered with a 70-µm cell strainer and 36% Percoll solution was used to separate cells. The cells were washed with cold PBS and filtered with a 40-µm cell strainer for staining. Spleen- and liver-infiltrating leukocytes were stained using the antibodies listed in **Supplementary Table S2** and analyzed *via* CytoFLEX LX.

### Statistical analysis

Statistical analyses were performed using SPSS (ver. 25.0). Continuous variables were expressed as the mean ± SD. One-way ANOVA was used to compare the NC, ConA, and CM groups. For *post-hoc* multiple comparisons, Bonferroni or Tamhane’s T2 was used to compare two groups according to the homogeneity of variance test. For correlation analysis between immune cells and AIH severity, Pearson correlation analysis was used. In all analyses, *p* < 0.05 was taken to indicate statistical significance.

## Results

### MenSCs ameliorated ConA-induced hepatitis

**Figure 1:**
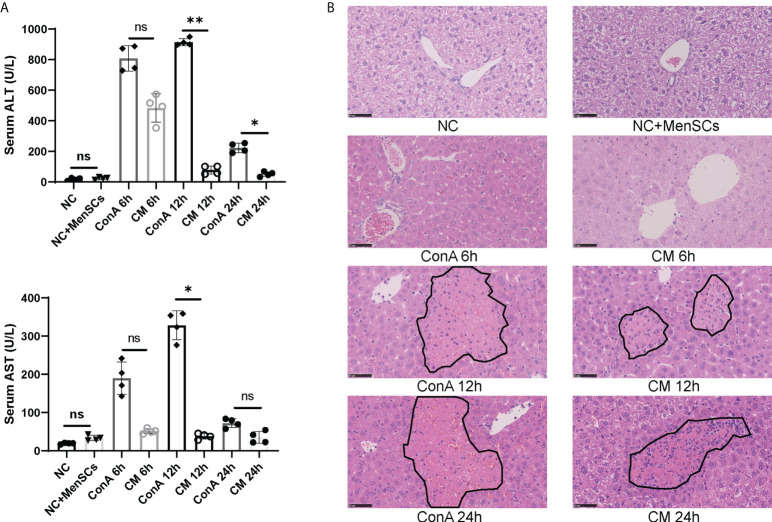
MenSCs transplantation ameliorated AIH mouse model. **(A)** Serum ALT and AST levels of ConA and CM groups at 6, 12, and 24 h and NC and NC+MenSCs groups (n = 4 per group; ns, not statistically significant, *p < 0.05, **p < 0.01). **(B)** Representative images of liver sections stained with H&E (n = 4 per group; black circles represent hepatocellular damage). Data are presented as mean ± SD.

ConA-induced hepatitis was used as AIH model to explore the therapeutic effects of MenSCs. The safety of MenSCs was examined by comparing ALT and AST in the NC+MenSCs and NC groups (**Figure 1A**). Serum ALT and AST levels in Con A group and CM group were measured at 6, 12, and 24 h, respectively. ConA could induce liver injury in mice 2 h after injection of ConA (25). In our study, ALT and AST peaked at 12 h and declined rapidly. In addition, the Con A group had higher ALT at 12 and 24 h and higher AST at 12 h than the CM group (**Figure 1A**). H&E staining also showed that hepatocytes injury in the CM group was lighter than that in the Con A group (**Figure 1B**). In addition, we measured human MCP-1 levels in mice serum that may play a role in the immune regulation of MSCs (22). The results showed that MenSCs could secret high levels of MCP-1 at NC+MenSCs and CM 6 h groups (**Supplementary Figure S1A**). Since MenSCs could rapidly improve Con A-induced hepatitis at 12 h, we chose 12 h as the most important time point in our further study.

### The overall CD45^+^ immune cells change in ConA and CM groups

**Figure 2:**
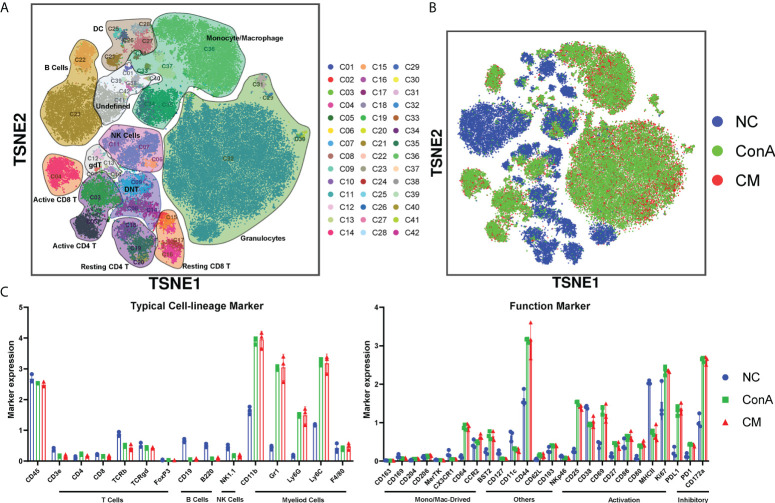
Overview of the CyTOF landscape of immune cells in liver samples. **(A)** t-SNE map colored by different subgroups. **(B)** t-SNE map colored by three groups. **(C)** Histograms displaying cell surface marker expression of typical cell lineage markers and functional markers, separately. Data are presented as mean ± SD.

**Figure 3:**
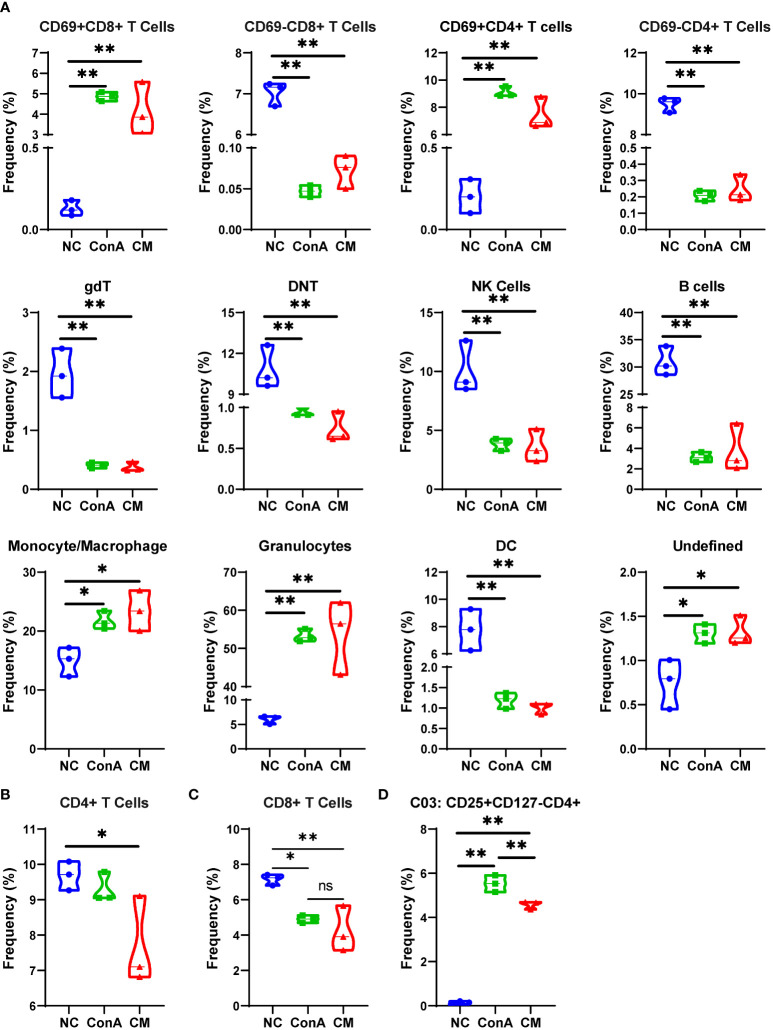
Major cells type analysis of different groups among immune cells. **(A)** The percentages of different groups in the NC, ConA, and CM groups among CD45^+^ immune cells in the liver tissue. **(B–D)** The cell frequency of CD4^+^ and CD8^+^ T cells and C03 cells in the NC, ConA, and CM groups among all immune cells in the liver tissue (n = 3 per group; ns, not statistically significant, *p < 0.05, **p < 0.01). Data are presented as mean ± SD.

We used a panel of 42 antibodies shown in **Supplementary Table S1** to characterize CD45^+^ immune cell population (**Supplementary Figures S2A, B**), and 42 different cell subgroups were identified from all nine liver samples (**Figure 2A**; **Supplementary Figure S2A**). Comparing the three groups in cell clustering (**Figure 2B**) and cell markers expression (**Figure 2C**), ConA could significantly alter the immune cells population and marker expression in the liver tissue, such as myeloid cells lineage markers, PD-L1, MHCII, and CD69. However, ConA and CM groups distributions and markers expression were similar in CD45^+^ immune cells subgroups (**Figure 2B**). We clustered the 42 subgroups into 12 large subpopulations, and their proportions and cluster distribution are shown in **Figure 3A** and **Supplementary Figures 3A, B**. The Granulocytes, monocytes/macrophages, and undefined subgroups were significantly upregulated by ConA, while CD4^+^, CD8^+^, gdT, (double negative T) DNT, NK cells, B cells, and DC were downregulated. The CM group displayed similar tendency with the ConA group (**Figures 3A-C**). In addition, CD69 is referred as a marker for early activated lymphocytes (26), and CD69^+^ Th and Tc cells could play important roles in liver injury of AIH (25). We found that ConA significantly increased CD69^+^ T cells among CD4^+^ and CD8^+^ subgroups (**Figure 3A**). Moreover, ConA upregulated the C03 subgroup, which represents a type of regulatory T cells (27), and MenSCs lessened this effect (**Figure 3D**).

### ConA and CM groups had similar alterations among T-cell subtypes

**Figure 4:**
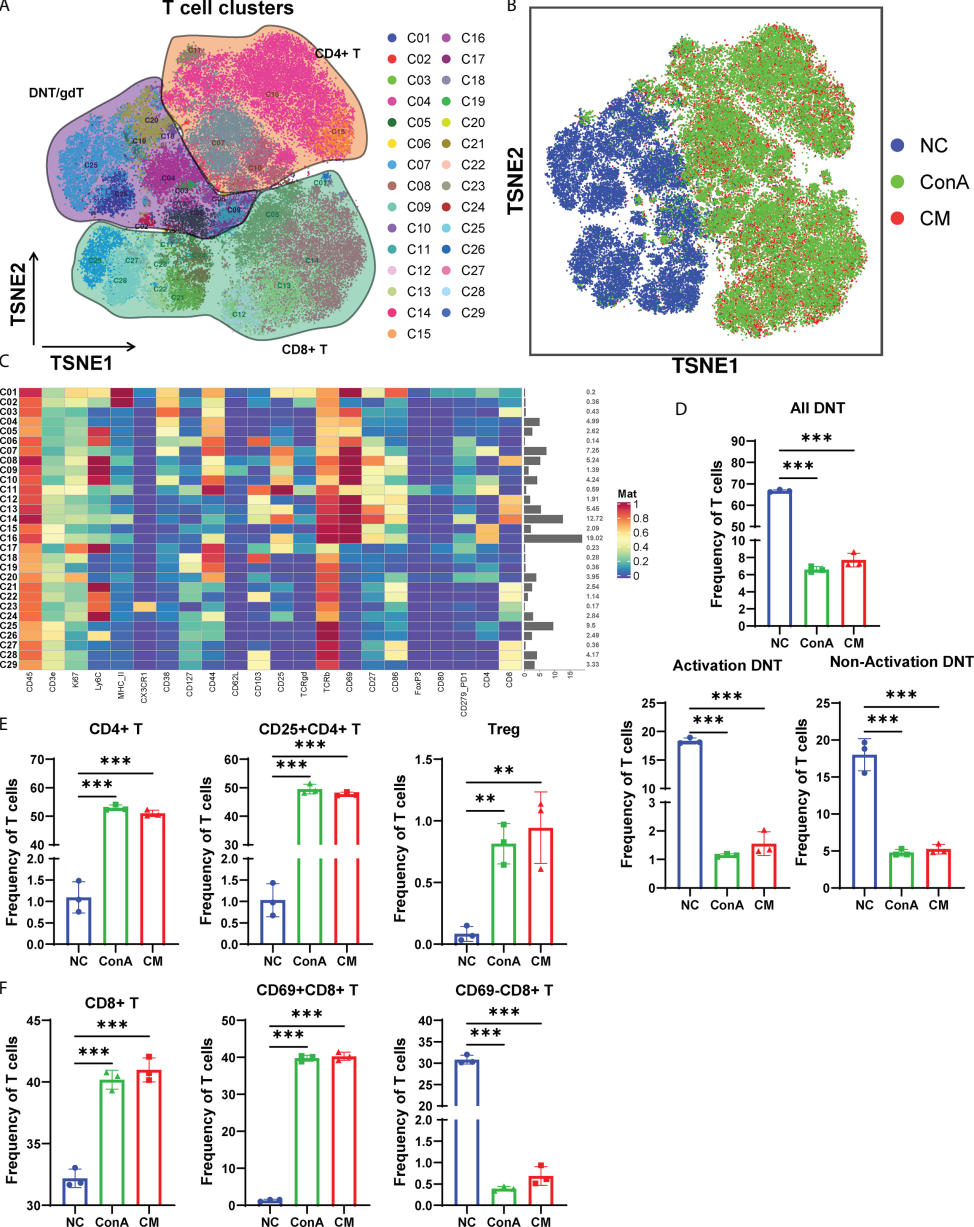
Overview of the CyTOF landscape of T cells in liver samples. **(A)** t-SNE map colored by different subgroups. **(B)** t-SNE map colored by three groups. **(C)** Heatmaps of cell surface markers expressed in T-cell subtypes. The proportions of **(D)** DNT; **(E)** CD4^+^, CD25^+^CD4^+^, and Treg; and **(F)** CD8^+^, CD69^+^CD8^+^ and CD69^−^CD8^+^ T-cell subtypes among the three groups (n = 3 per group; ns, not statistically significant, **p < 0.01, *** *p* < 0.001). Data are presented as mean ± SD.

Next, we gated the CD3^+^ cell population among CD45^+^ cells to further explore the changes in T cell subsets in-depth (**Figure 4A**). The t-SNE map of T-cell clustering displayed that the NC group was significantly different from the other two groups (**Figure 4B**). The NC group mainly concentrated on DNT/gdT, and the proportion of CD8^+^ T cells is relatively small, but the ConA and CM groups are almost CD4^+^ T and CD8^+^ T cells (**Figures 4A, B**). In addition, the heatmap showed that the CD69^+^ subsets were mainly gathered in ConA and CM groups (**Figure 4C**). By focusing on DNT, we found that no matter activation DNT or non-activation DNT was reduced by ConA administration (**Figure 4D**). The percentages of CD4^+^ T, CD25^+^CD4^+^ T, and Treg were higher in ConA and CM groups (**Figure 4E**) and the CD8^+^ and CD69^+^CD8^+^ T cells (**Figure 4F**).

### MenSCs reduced M1 frequency in macrophages in ConA-induced hepatitis

**Figure 5:**
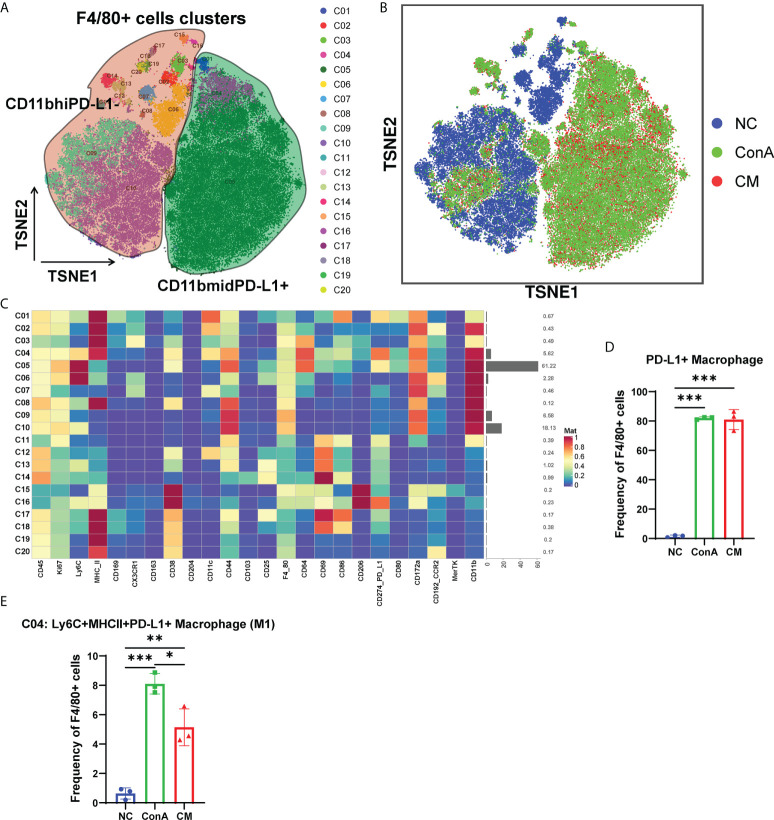
Overview of the CyTOF landscape of F4/80^+^ cells in liver samples. **(A)** t-SNE map colored by different subgroups. **(B)** t-SNE map colored by three groups. **(C)** Heatmaps of cell surface markers expressed in F4/80^+^ cell subtypes. The proportions of **(D)** PD-L1^+^ macrophages and **(E)** the C04 subtype among the three groups in F4/80^+^ cells. (n = 3 per group; *p < 0.05, **p < 0.01, ***p < 0.001). Data are presented as mean ± SD.

Since macrophages could be activated 1 h of ConA injection (28). Then, we gated the F4/80^+^ cell population among CD45^+^ (**Figure 5A**). t-SNE map showed that the NC group could be separated from ConA and CM groups (**Figure 5B**). NC mainly concentrated on CD11bhiPD-L1^−^ cluster, and ConA and CM groups mainly contained CD11bmidPD-L1^+^ (**Figures 5A, B**). The heatmap displayed marker expression of F4/80^+^ cell subtypes (**Figure 5C**). PD-L1^+^ could be expressed in myeloid immune cells and bind to PD-1 to regulate T-cell suppression. The interaction in macrophages could increase spontaneous macrophage proliferation, survival, and activation (29). We checked the PD-L1^+^ macrophages proportion (**Figure 5D**), which contains C01, C04, and C05 clusters in **Figure 5C**, and ConA and CM groups had a much higher frequency than the NC group. Among the PD-L1^+^ macrophages, we further analyzed the frequency of Ly6C^+^CXCR3^−^MHCII^+^ macrophages (M1), and ConA significantly increased the percentage of C04 cells compared with that in the NC group, while MenSC treatment reduced this increase (**Figure 5E**).

### Active CD4^+^ T cells and macrophages were positively correlate with ConA-induced hepatitis severity

To further explore the correlation between immune cells and the severity of ConA-induced hepatitis, we analyzed the association of all identified immune cell clusters with ALT and AST levels at 12 h in ConA, CM group, and NC group by Pearson correlation analysis. gdT (C02), CD69^+^CD4^+^ T (C03, C05, **Supplementary Figure S4**), and DC (C28) of CD45^+^ immune cells were positively correlated with mice serum ALT levels (**Table 1**; **Supplementary Table S3**). CD45^+^CD4^+^ immune cell clusters were not significantly associated with ALT and AST levels (**Supplementary Table S4**). PD-L1^+^ macrophages (C01 and C04) of CD45^+^F4/80^+^ immune cells were positively related with ALT and AST levels (**Supplementary Table S5**).

**Table 1:** Correlation of CD45^+^ immune cells subpopulations with ALT and AST.

	ALT			AST		
	Pearson correlation	Sig. (2-tailed)	N	Pearson correlation	Sig. (2-tailed)	N
Undefined	0.441	0.234	9	0.418	0.263	9
gdT	−0.518	0.153	9	−0.505	0.165	9
CD69^+^CD4^+^ T	.686^*^	0.041	9	0.667^*^	0.050	9
CD69^+^CD8^+^ T	0.640	0.064	9	0.623	0.073	9
NK cells	−0.482	0.189	9	−0.479	0.192	9
DNT	−0.531	0.141	9	−0.517	0.154	9
CD69^−^CD8^+^ T	−0.556	0.120	9	−0.541	0.132	9
CD69^−^CD4^+^ T	−0.556	0.120	9	−0.542	0.132	9
DC	−0.519	0.152	9	−0.508	0.163	9
B cells	−0.564	0.114	9	−0.552	0.124	9
Monocyte/macrophage	0.341	0.369	9	0.335	0.378	9
Granulocytes	0.530	0.143	9	0.519	0.152	9

* Sig. (2-tailed) < 0.05;

### Analysis of the immune cells in the liver tissue and spleen by conventional flow cytometry

**Figure 6:**
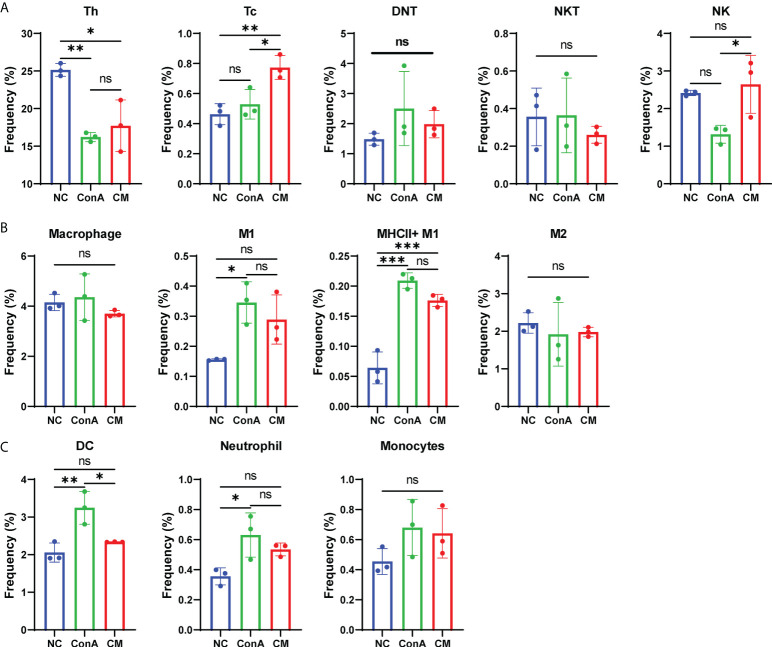
Changes in immune cells in mouse spleen tissue. **(A)** The frequency of Th, Tc, DNT, NKT, and NK cells; **(B)** the frequency of macrophage, M1, MHCII^+^ M1, and M2 cells; **(C)** the frequency of DC cells, neutrophil, and monocytes among NC, ConA, and CM groups (n = 3 per group; ns, not statistically significant, *p < 0.05, **p < 0.01, ***p < 0.001). Data are presented as mean ± SD.

In our previous study, we found that MenSCs could reside in the spleen (24). As the spleen is an important immune organ, we wanted to find the immune cells changing in the spleen and liver by conventional flow cytometry (**Supplementary Figures S5A, B**). The CM group had fewer Tc cells than the ConA group in the liver tissue (**Supplementary Figure S6A**), while CM had a higher frequency of Tc cell in the spleen (**Figure 6A**). MenSCs could recover the elevated NKT induced by ConA in the liver (**Supplementary Figure S6A**). Macrophage and M1 and M2 frequency were elevated by ConA, and MenSCs could reduce the frequency of macrophage and MHC^+^ M1 cells in the liver (**Supplementary Figure S6B**). The ConA group had higher M1 and MHCII^+^ M1 cells in the spleen than the NC group (**Figure 6B**). ConA had fewer DC cells in the liver (**Supplementary Figure S6C**) and higher DC cells in the spleen (**Figure 6C**) than the NC group. In addition, ConA and CM groups had a higher frequency of neutrophil and monocytes in the liver than the NC group (**Supplementary Figure S6C**).

### MenSCs alternated the inflammatory cytokines in ConA-induced hepatitis

**Figure 7:**
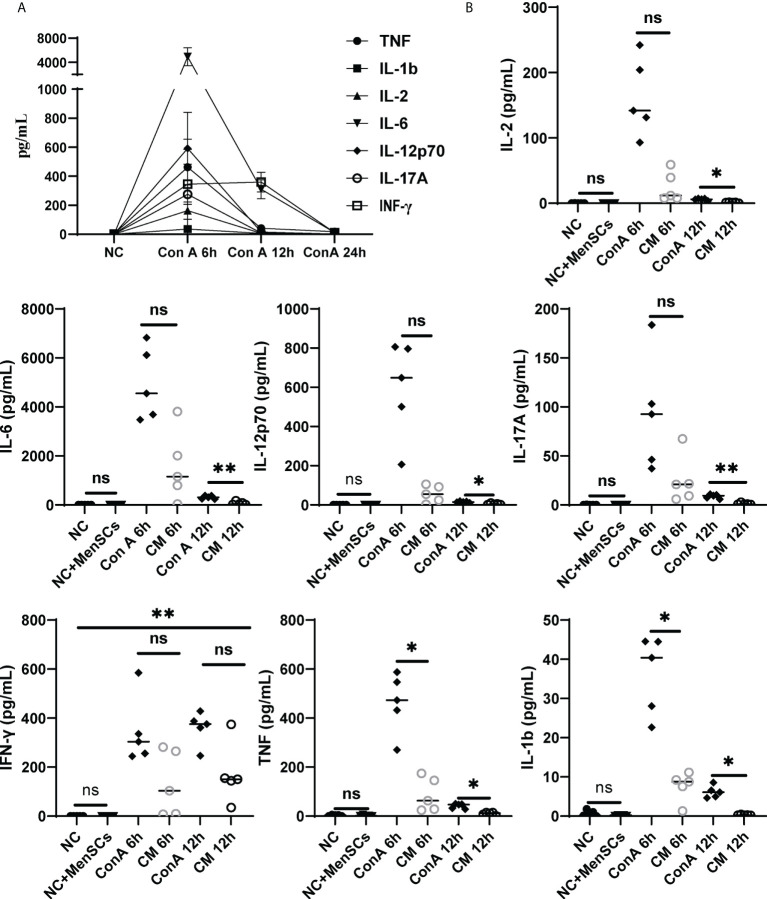
Changes in inflammatory factors among the NC, ConA, and CM groups at 6 and 12 (h) **(A)** Inflammatory factors (TNF-α, IL-17A, IL-12p70, IL-6, IL-2, IL-1b, and IFN-γ) in ConA group serum (n = 5 per group at 6, 12, and 24 h). **(B)** The inflammatory factors (TNF-α, IL-1b, IFN-γ, IL-17A, IL-17A, IL-2, and IL-12p70) were tested in the ConA group and CM group at 6, 12, and 24 h and in the NC group and NC+MenSCs group at 24 h (n = 5 per group; ns, not statistically significant, *p < 0.05, **p < 0.01). Data are presented as mean ± SD.

Furthermore, we detected pro-inflammatory cytokines TNF, IL-1β, IL-12p70, IFN-γ, IL-6, IL-2, and IL-17A corresponding with ConA-induced hepatitis and found that pro-inflammatory factors all rise at 6 h after ConA injection (**Figure 7A**). Then, we compared cytokines among NC, NC+MenSCs, ConA, and CM groups at 6 and 12 h. We showed that MenSCs could significantly reduce the production of TNF and IL-1β induced by ConA injection at 6 h, and IL-17A, IL-6, IL-12p70, IL-2, TNF, and IL-1β at 12 h (**Figure 7B**). In addition, we measured the anti-inflammation cytokine IL-10 in mice serum. MenSCs could significantly upregulate the IL-10 level of the CM 6 h group (**Supplementary Figure S7A**).

## Discussion

AIH is described as progressive inflammation of hepatocytes mediated by autoimmune response to liver parenchyma. The occurrence of AIH is widespread worldwide and can occur at all ages in genetically susceptible individuals (5). In 1992, Tiegs et al. established an acute immune hepatitis model by intravenously injecting ConA (30). ConA in one of the lectins mostly binds to the hepatocyte membrane to induce liver injury *via* T cells and macrophages (28). The mechanisms of ConA mouse model have been studied for several years, and the main standpoints were focused on active T cells, NKT, and macrophages (28, 31, 32). MenSCs could be an effective treatment for cholestatic liver injury by regulating β-catenin expression (24) and antifibrotic capacity in chronic liver diseases (17). We found that MenSCs could significantly rescue ConA mice at 15 mg/kg dosage in serum ALT, AST levels, and H&E staining. However, most studies focused on the mechanisms of apoptosis, autophagy, and pyroptosis of the ConA model (33–35); the global view of ConA model’s immune alterations remained unclear. We used CyTOF and conventional flow cytometry to comprehensively clarify ConA and CM groups’ immune cells changes and CBA to check the cytokines changing.

CD69 is referred to as a marker for early activated lymphocytes (26). We noted that ConA administration could give a prominent increase in CD69^+^ T cells among all CD45^+^ leukocytes, which were supposed as active T cells (36). Combining the results of CyTOF and conventional flow cytometry, we found that CD69^+^ T cells were notably elevated by ConA, and CD69^−^ T cells were significantly reduced, with total CD4^+^ T cells showing similar percentage between NC and ConA group, and total CD8^+^ T cells were significantly reduced by ConA injection. Thus, it is possible that ConA injection activate the T cells, and Tc cells were consumed, while MenSCs could not inhibit the activation of T cells and Tc cells depletion. Murasko et al. found that ConA could stimulate T cells, especially CD69^+^ T cells, from the spleen to proliferation and expansion *in vitro* (37). However, our flow cytometry data of splenocytes showed that Th cells were less in ConA and CM groups than NC group with alike frequency of Tc cells between NC and ConA groups in splenocytes. Tc cells in the spleen of the CM group were much higher than that in the NC and ConA groups, while that in the liver tissue were much less.

NKT and DNT are parts of the atypical T cells that account for certain percentages in mice liver. Previous studies have shown that NKT is critically involved in the process of ConA-induced hepatitis *via* NKT-derived proinflammation response and cytotoxicity (32, 38). Our flow cytometry data also displayed that ConA could increase NKT in the liver tissue more than NC and MenSCs groups. In addition, we found that DNT was significantly less in ConA and CM groups than NC group no matter in all CD45^+^ leukocytes or CD45^+^CD3^+^ T cell clusters. There are few reports of DNT and ConA by far (39), and MenSCs showed no significant effect on DNT as well.

Previous research showed that Kupffer cells have vital importance to ConA-induced hepatitis with a tough TNF secretion ability (40, 41). Seki et al. found that after ConA injection, liver macrophages were activated, and the balance of M1/M2 shifted to M1 significantly (40). Our data also displayed that ConA could remarkably promote macrophages and M1 percentages in the liver tissue than the NC group, and MenSCs could conspicuously inhibit the process. As Tiegs et al. found that specifically blocking macrophages *via* liposome-encapsulated dichloromethylene-bisphosphonate could dominantly inhibit ConA effects (41), MenSCs may have a vital importance in mediating ConA-induced hepatitis by inhibiting macrophages activation and M1 polarization. In addition, MenSCs could downregulate proinflammatory cytokines levels, which are important in AIH (28).

Above all, we conclude that ConA can affect liver leukocyte alterations in nearly all clusters that we detected. The t-SNE can perfectly separate the ConA and NC groups. We focused on Th, Tc, NKT, and macrophages, and MenSCs may mostly affect ConA-induced macrophages to effect. MenSCs-based therapy has a great potential for AIH clinical manifestation, so further elucidation of its mechanisms is valuable. In addition, induced pluripotent stem-cell-derived MSCs (iPSCs-MSCs) have received increasing attention as a source of mesenchymal stem cells due to their higher proliferative capacity without significant loss of self-renewal potential (42). iPSCs-MSCs were found to be safe and well-tolerated in refractory graft-versus-host disease clinical trials (43). MenSCs-based therapy requires further studies and verification for clinical usage.

## Data availability statement

The raw data supporting the conclusions of this article will be made available by the authors, without undue reservation.

## Ethics statement

This study was reviewed and approved by The tab of Animal Care Committee of the Animal Experimental Ethical Inspection of the First Affiliated Hospital, College of Medicine, Zhejiang University (permit number: 2020-1505).

## Author contributions

The author’s responsibilities were as follows: LL, FZ, SZ, and LF designed the study. QHL, LZ, NM, and FZ participated in all animal work. QL cultured MenSCs. FZ, LF, ST, and LX performed CyTOF and analyzed the data. All authors contributed to the manuscript and approved the submitted version.

## Funding

This study was funded by the Science Fund for Creative Research Groups of the National Natural Science Foundation of China (No. 81721091) and The Independent Task of State Key Laboratory for Diagnosis and Treatment of Infectious Diseases.

## Conflict of interest

The authors declare that the research was conducted in the absence of any commercial or financial relationships that could be construed as a potential conflict of interest.

## Publisher’s note

All claims expressed in this article are solely those of the authors and do not necessarily represent those of their affiliated organizations, or those of the publisher, the editors and the reviewers. Any product that may be evaluated in this article, or claim that may be made by its manufacturer, is not guaranteed or endorsed by the publisher.
