# Supplementary material for: Comprehensive immune cell analysis of human menstrual-blood-derived stem cells therapy to concanavalin A hepatitis
Source: Front Immunol. 2022 Sep 29;13:974387. doi: 10.3389/fimmu.2022.974387 (PMC9559565; doi:10.3389/fimmu.2022.974387)
Supplement: Supplementary file 1 [file DataSheet_1.docx]

**Supplementary Figures**


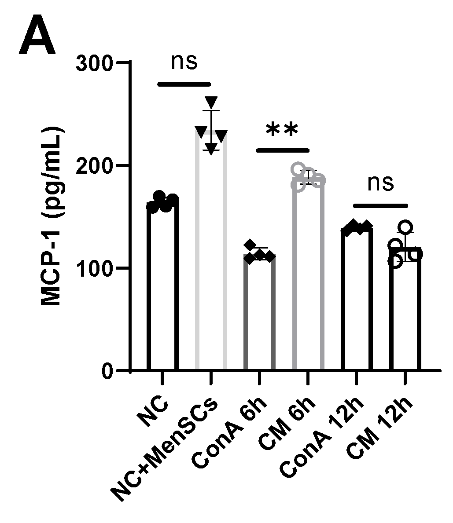


**Supplementary Figure 1** Serum MCP-1 levels in ConA and CM groups at 6 h, 12 h, and NC group. (n = 4 per group, **p* < 0.05, ***p* < 0.01). Data were represented as Mean ± SD.


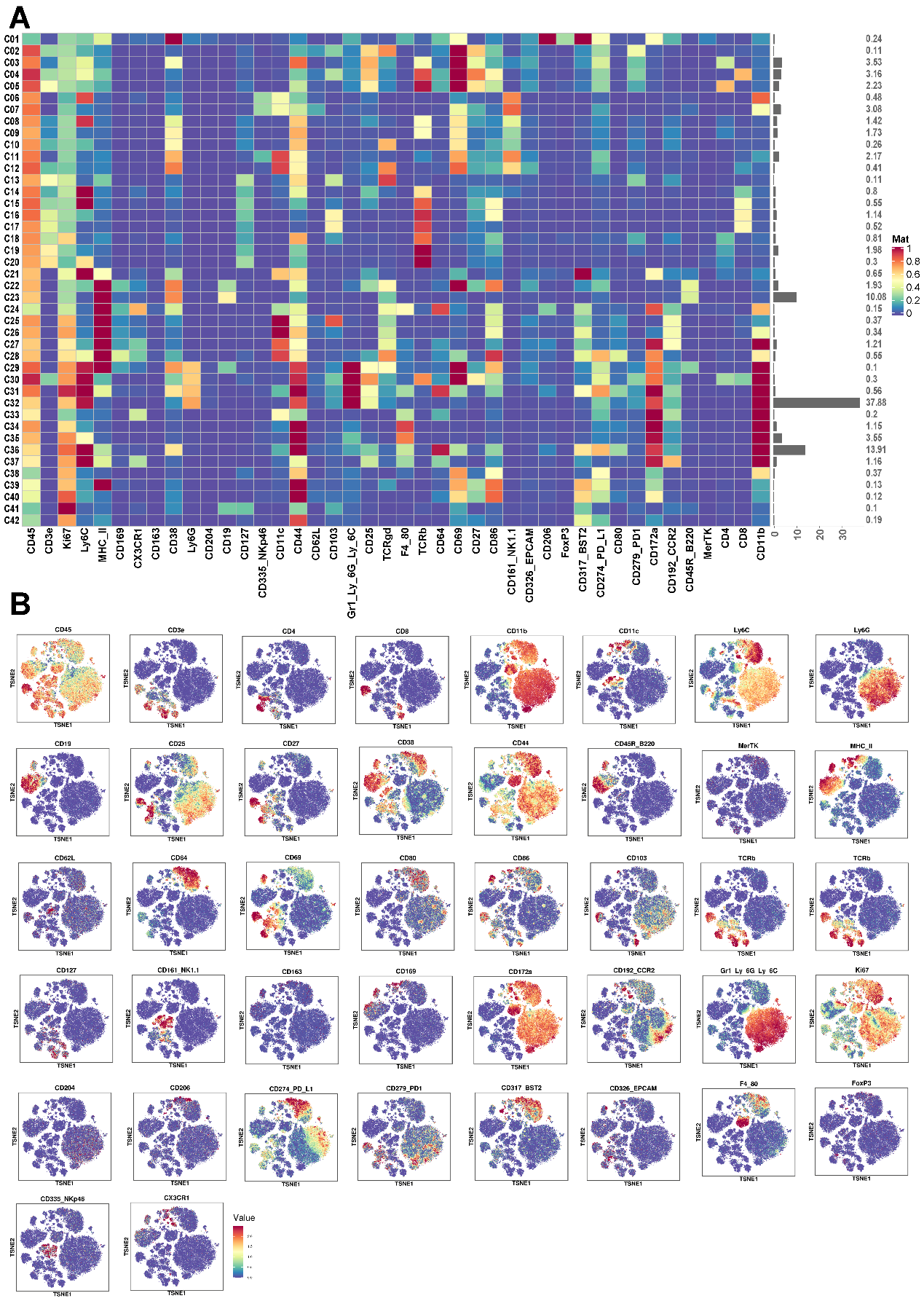


**Supplementary Figure 2** Overview of cell markers expressed in CD45^+^ immune cells. **(A)** Heat maps; **(B)** t-SNE maps displayed the expression of cell markers in CD45^+^ immune cells.


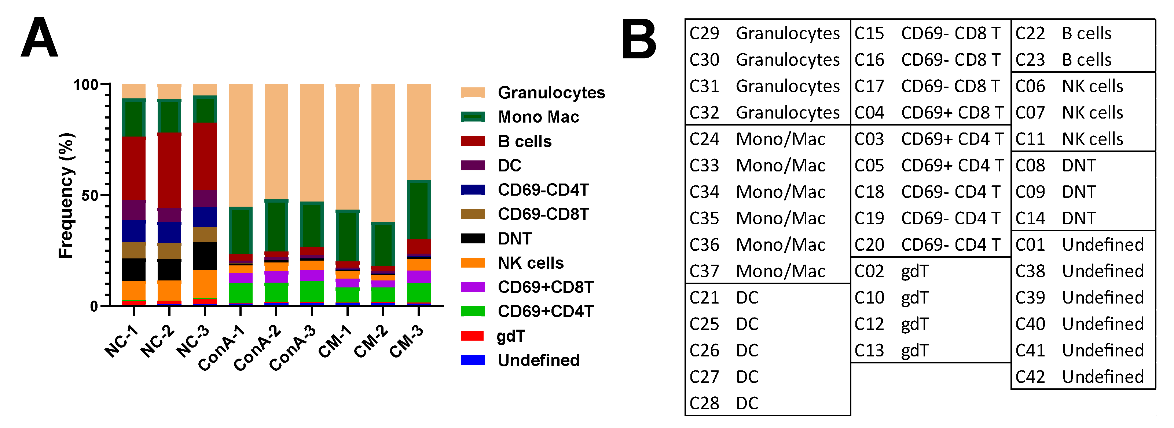


**Supplementary Figure 3** Major cell types analysis of all samples in CD45^+^ immune cells. **(A)** Stacked bar maps of subpopulation proportions among nine samples. **(B)** The cluster-distribution table of major cell types. Mono, monocyte; Mac, macrophage.


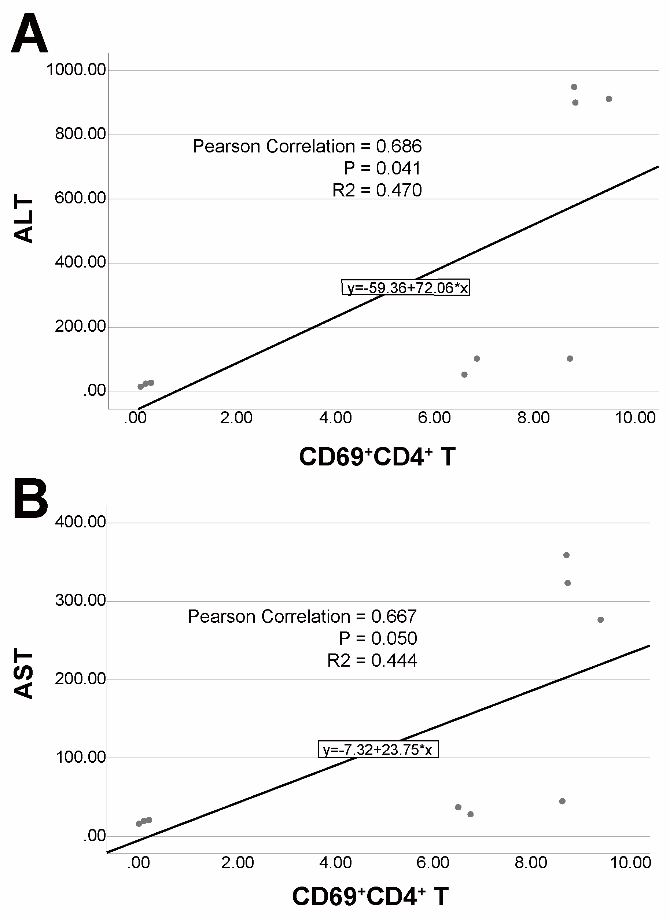


**Supplementary Figure 4** Correlation of CD45^+^ immune cells subpopulations with AIH severity. (A) ALT and (B) AST.


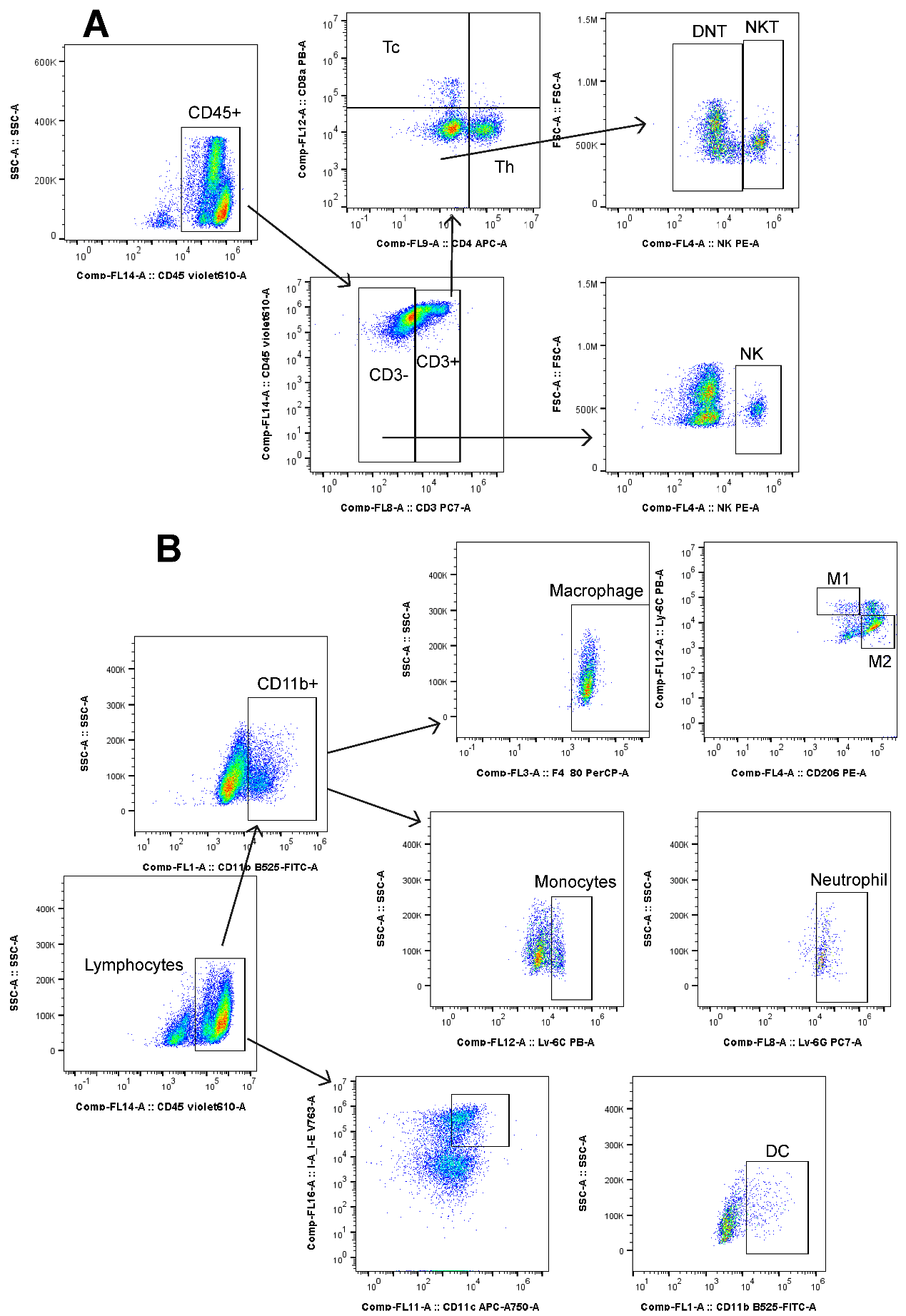


**Supplementary Figure 5** Two flow cytometry panels of liver and spleen tissues. The flow cytometry’s flow chart of **(A)** lymphocytes; **(B)** myeloid cells.


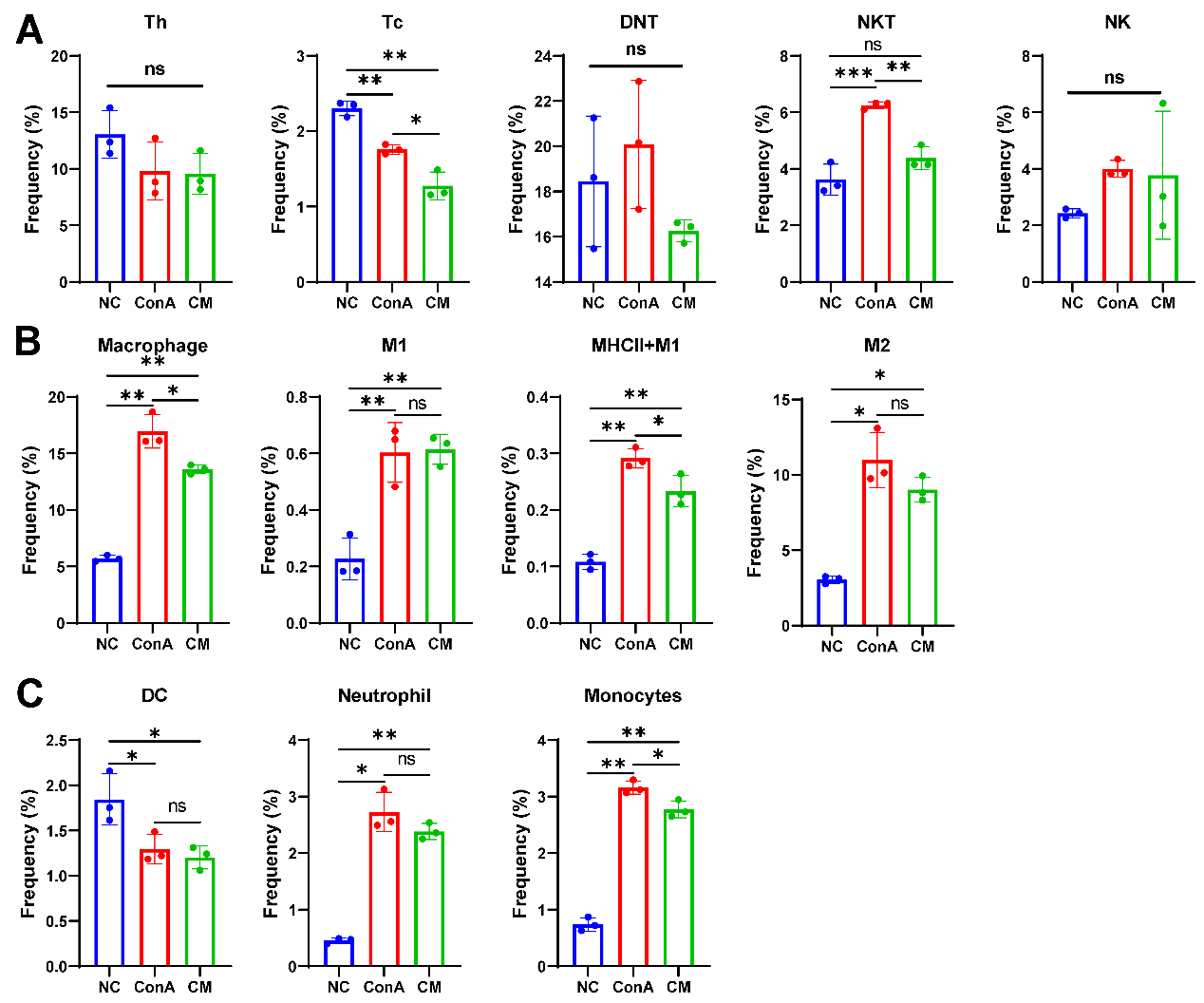


**Supplementary Figure 6** Changes of immune cells in mouse liver tissue. **(A)** The frequency of Th, Tc, DNT, NKT and NK cells; **(B)** The frequency of macrophage, M1, MHCII^+^ M1 and M2 cells; **(C)** The frequency of DC cells, neutrophil, and monocytes among three groups. (n = 3 per group, **p* < 0.05, ***p* < 0.01). Data were represented as Mean ± SD.


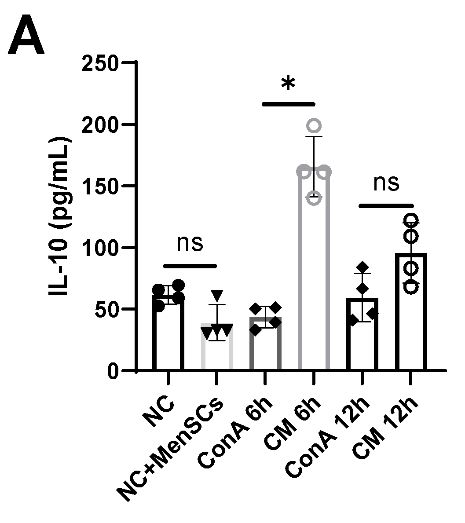


**Supplementary Figure 7** Serum IL-10 levels in ConA and CM groups at 6 h, 12 h, and NC group. (n = 4 per group, **p* < 0.05, ***p* < 0.01). Data were represented as Mean ± SD.
